# Embedding Quantum into Classical: Contextualization vs Conditionalization

**DOI:** 10.1371/journal.pone.0092818

**Published:** 2014-03-28

**Authors:** Ehtibar N. Dzhafarov, Janne V. Kujala

**Affiliations:** 1 Department of Psychological Sciences, Purdue University, West Lafayette, Indiana, United States of America; 2 Department of Mathematical Information Technology, University of Jyväskylä, Jyväskylä, Finland; University of Nottingham, United Kingdom

## Abstract

We compare two approaches to embedding joint distributions of random variables recorded under different conditions (such as spins of entangled particles for different settings) into the framework of classical, Kolmogorovian probability theory. In the contextualization approach each random variable is “automatically” labeled by all conditions under which it is recorded, and the random variables across a set of mutually exclusive conditions are probabilistically coupled (imposed a joint distribution upon). Analysis of all possible probabilistic couplings for a given set of random variables allows one to characterize various relations between their separate distributions (such as Bell-type inequalities or quantum-mechanical constraints). In the conditionalization approach one considers the conditions under which the random variables are recorded as if they were values of another random variable, so that the observed distributions are interpreted as conditional ones. This approach is uninformative with respect to relations between the distributions observed under different conditions because any set of such distributions is compatible with any distribution assigned to the conditions.

## Introduction

### Joint Distributions and Stochastic Unrelatedness

Many scientific problems, from psychology to quantum mechanics, can be presented in terms of *random outputs* of some system recorded under various *conditions*. According to the principle of *Contextuality-by-Default* [1]–[4], when applying Kolmogorov's probability theory (KPT) to such a problem, random variables recorded under different, mutually incompatible conditions should be viewed as *stochastically unrelated* to each other, i.e., possessing no joint distribution. They can always be “sewn together” as part of their theoretical analysis, but joint distributions are then *imposed* on them rather than derived from their identities. In this paper we discuss two possible approaches to the foundational issue of “sewing together” stochastically unrelated random variables. We call these approaches *contextualization* and *conditionalization*. The former takes the Contextuality-by-Default principle as its departure point and is, in a sense, its straightforward extension; in the latter, Contextuality-by-Default is obtained as a byproduct.

To understand why the Contextuality-by-Default principle is associated with either of these two approaches, one should first of all abandon the naive notion that in KPT any two random variables have a joint distribution uniquely determined by their definitions. A random variable is a measurable function on a probability space, and the notion of a single probability space for all possible random variables (or, equivalently, the notion of a single random variable of which all other random variables are functions) is untenable. It contradicts the commonly used KPT constructions. (In this discussion we impose no restrictions on the domain and codomain probability spaces. A random variable therefore is understood in the broadest possible way, including random vectors, random functions, random sets, etc. We will avoid, however, the use of general measure-theoretic formalism.)

One of them is, given any set, to construct a random variable whose range of possible values coincides with this set. A probability space on which all such random variables were defined would have to include a set of cardinality exceeding that of all possible sets, an impossibility.

Another commonly used construction is, given a random variable, to introduce another random variable that has a given distribution and is stochastically independent of the former. The use of this construction contradicts even the notion of a jointly distributed set of all variables with a particular distribution [2], say, the set 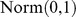 of all standard-normally distributed random variables. Indeed, if all random variables in 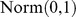 were jointly distributed, they would all be presentable as functions of some random variable 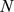, the identity function on the probability space on which the random variables in 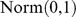 are defined. Choose now a standard-normally distributed random variable 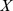 so that it is independent of 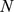. Then it is also independent of any 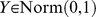. Since 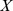 cannot be independent of itself, 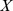 cannot belong to 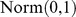. At the same time, 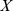 must belong to 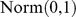 due to its distribution.

Short of imposing on KPT artificial constraints (such as an upper limit on cardinality of the random variables' ranges), these and similar contradictions can only be dissolved by allowing for stochastically unrelated random variables defined on different probability spaces (see Ref. [5] for how this can be built into the basic set-up of probability theory). The principle of Contextuality-by-Default eliminates guesswork from deciding which random variables are and which are not jointly distributed. Irrespective of how one defines a system with random outputs and identifies the conditions under which these outputs are recorded, the outputs are jointly distributed if they are recorded under one and the same set of conditions; if they are recorded under different, mutually exclusive conditions, they are stochastically unrelated.

### Two Approaches

Contextualization and conditionalization differ in how they “sew together” stochastically unrelated random variables. To demonstrate these differences on a simple example, let 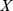 and 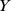 be random variables with 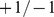 values, so that their distributions are determined by 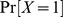 and 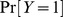, respectively. Let 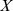 and 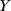 be recorded under mutually exclusive conditions.

In contextualization (the approach we proposed in Refs. [1]–[4]), one first invokes the Contextuality-by-Default principle to treat 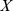 and 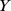 as stochastically unrelated random variables. A “sewing together” of 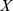 and 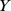 consists in *probabilistically coupling* them [6], i.e., presenting them as functions of a single random variable. Put differently (but equivalently), we create a random variable (vector) 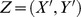 such that 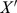 is distributed as 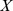 and 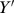 is distributed as 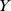. The variables 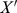
and 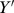 are jointly distributed (otherwise 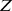 would not be called a random variable, or a random vector), but this distribution is not unique. Thus, 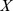 and 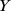 can always be coupled as stochastically independent random variables, so that
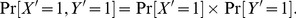
(1)


They can also be coupled as identical random variables,
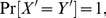
(2)but only if 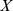 and 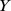 are distributed identically,
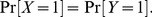
(3)


There can, in fact, be an infinity of couplings, constrained only by
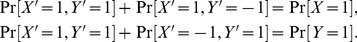
(4)


In conditionalization, one creates a random variable 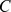 with two possible values corresponding to the two sets of conditions under which one records 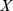 and 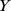, respectively. Then one defines a random variable 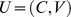, such that the conditional distribution of 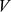 given 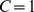 is the same as the distribution of 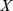, and the conditional distribution of 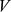 given 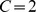 is the same as the distribution of 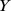. In other words,
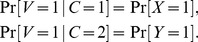
(5)


The principle of Contextuality-by-Default here does not have to be invoked explicitly, but it is adhered to anyway: the random variable 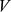 is related to conditions under which it is recorded, and 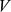 conditioned on 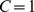 clearly has no joint distribution with 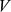 conditioned on 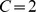.

Conditionalization can also be implemented in more complex constructions, such as the one proposed in Ref. [7]. In our example, this construction amounts to replacing 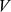 with two random variables, 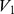 and 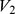, and “coordinating” their possible values with the values of 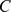. Thus, one can make 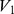 and 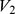 binary, 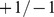, and define the conditional distributions by
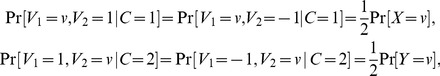
(6)where 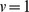 or 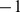. For 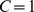, as we see, the “relevant” output is 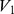, and the probabilities of its values 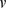 are simply evenly divided between the two possible values of the “irrelevant” output 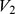 (and for 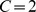, 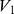 and 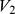 exchange places).

We argue in this paper that only contextualization serves as a useful tool for classifying and characterizing different types of systems involving random outputs that depend on conditions (e.g., classical-mechanical vs quantum-mechanical systems). Conditionalization, both in its simplest and modified versions, is always applicable but uninformative.

### Quantum Entanglement

Our analysis pertains to any input-output relations, as considered in Refs. [1]–[3], [8]–[11]. The relations can be physical, biological, behavioral, social, etc. For the sake of mathematical transparency, however, we confine our consideration to the canonical quantum-mechanical paradigm [12] involving two entangled particles, “Alice's” and “Bob's.” Alice measures the spin of her particle in one of two directions, 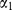 or 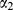 (values of the first input), and Bob measures the spin of his particle in one of two directions, 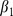 or 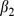 (values of the second input). Each pair of measurements is therefore characterized by one of four possible combinations of input values 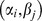, and it is these combinations that form the four *conditions* in this example. The spins recorded in each trial are realizations of random variables (outputs) 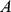 and 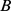, which, in the simplest case of spin-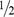 particles, can attain two values each: “up” or “down” (encoded by 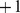 and 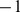, respectively).

Aside from simplicity, another good reason for using this example is that it relates to the problem of great interest in the foundation of physics: in what way and to what an extent one can embed joint probabilities of spins in entangled particles into the framework of KPT? It may seem that this question was answered by John Bell in his classical papers [13], [14], and that the answer was: KPT is not compatible with the joint distributions of spins in entangled particles. However, in Bell's analysis and its subsequent elaborations [15], [16] the use of KPT is constrained by an added assumption that has nothing to do with KPT. Namely, the implicit assumption in these analyses is that of “noncontextuality”:

a spin recorded in Alice's particle is a random variable uniquely identified by the measurement setting (spatial axis) for which it is recorded (and analogously for Bob's particle).

In other words, the spin recorded by Alice for settings 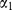 and 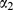 are different random variables 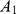 and 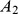, but the identity of either of them does not depend on whether Bob's setting is 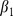 or 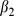 (and analogously for Bob's random variables 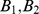 corresponding to 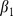 and 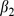). For well-established reasons (discussed in detail below), this makes a Kolmogorovian account of quantum entanglement impossible.

However, according to the Contextuality-by-Default principle, if one applies it to the Alice-Bob paradigm,

any two random variables recorded under mutually exclusive conditions are labeled by these conditions and considered stochastically unrelated.

Alice's spin values recorded under the condition 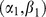 cannot *co-occur* with the spin values recorded by her under the condition 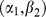, even though 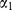 is the same in both conditions. Therefore the identity of the spin she measures under 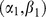 should be viewed as different from the identity of the spin she measures under 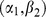.

This leads one to the double-indexation of the spins,
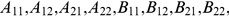
(7)


where 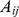 and 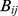 are the measurements by Alice and Bob, respectively, recorded under the condition 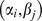, 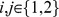. This vector of random variables cannot be called a *random vector* (or random variable, as we use the term broadly), because its components are not jointly distributed. Thus, 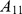 and 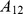, or 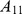 and 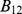, are recorded under mutually exclusive conditions, so they do not have jointly observed realizations. But the outputs 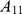 and 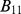, being recorded under one and the same condition 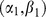, are jointly distributed, i.e., the joint probabilities for different combinations of co-occurring values of 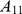 and 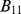 are well-defined. The situation is summarized in the following diagram:
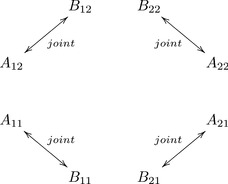
(8)


### Contextuality and No-Signaling

Why do we speak of “contextuality” and “noncontextuality”? The terms come from quantum mechanics (see, e.g., Refs. [17]–[21]), although it is not always clear that they are used in the same meaning as in the present paper. In the Alice-Bob paradigm with two spin-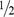 particles, the (marginal) distribution of Alice's measurement 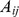 does not depend on Bob's setting 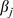, nor does the distribution of Bob's measurement 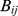 depend on 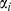:
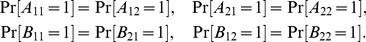
(9)


This is known as the *no-signaling* condition [22]: Alice, by watching outcomes of her measurements, is not able to guess Bob's settings, and vice versa. If the two particles are separated by a space-like interval, violations of no-signaling would contravene special relativity (and imply the “spooky action at a distance,” in Einstein's words).

Nevertheless, in KPT, 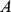 cannot be indexed by 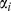 alone, nor can 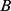 be indexed by 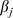 alone.

The logic forbidding single-indexation of the spins, 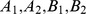, is simple [4]. Since, for any 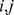, the random variables 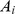 and 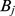 are jointly distributed, they are defined on the same probability space. Applying this consideration to 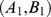, 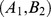, and 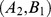, we are forced to accept that all four random variables, 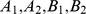, are defined on one and the same probability space. The existence of this joint distribution, however, is known to be equivalent to Bell-type inequalities (see below), known not to hold for entangled particles.

Therefore, in perfect compliance with the Contextuality-by-Default principle, we are forced to use the double indexation (7). We can say that while 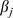 does not influence 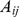 “directly” (which would be the case if 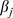 could affect the distribution of 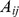), it generally creates a “context” for 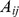. The context makes 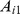 and 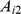 two different random variables with one and the same distribution, rather than one and the same random variable. (Analogous reasoning applies to 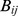 in relation to 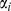.)

It should not, of course, come as a surprise that different random variables can be identically distributed. After all, it is perfectly possible that the distributions of Alice's spins for 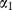 and 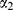 are identical too, and this would not imply that they are one and the same random variable. Within the framework of KPT, the difference between 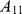 and 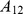 is essentially the same as the difference between 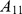 and 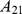: in both cases we deal with stochastically unrelated random variables, the only difference being that in the former pair, unlike in the latter one, the no-signaling condition forces the two random variables to be identically distributed. The notion of contextuality, however, does require broadening of one's thinking about how one decides that some empirical observations are and some are not realizations of one and the same random variable, as understood in KPT [2], [3].

## Theory

### Contextualization and Couplings

Contextualization is a straightforward extension of the Contextuality-by-Default principle. The latter creates the eight random variables in (7), and the contextualization approach consists in directly imposing a joint distribution on them. This can, of course, be done in infinitely many ways. Any random variable
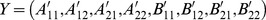
(10)such that, for any 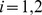 and 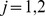,
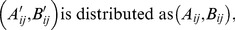
(11)is called a (probabilistic) *coupling* for (7) [6]. The fact that 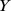 in (10) is referred to as a *random variable* (or random vector) implies that the components of 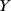 are jointly distributed, i.e., there is a joint probability assigned to each of the 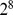 combinations of values for 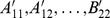.

The set of all possible couplings (10) for (7) is generally different for different distributions of the pairs 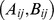. However, it always includes the coupling 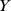 in which the pairs 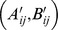 are stochastically independent across different 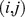. This coupling is referred to as an *independent coupling*. Its universal applicability leads to the common confusion of stochastic unrelatedness with stochastic independence. But stochastic independence is merely a special property of a joint distribution.

The non-uniqueness of the coupling (10), rather than being a hindrance, can be advantageously used in theoretical analysis. According to the *All-Possible-Couplings* principle formulated in Refs. [2], [3],

a set of stochastically unrelated random variables is characterized by the set of all possible couplings that can be imposed on them, with no couplings being a priori privileged.

Thus, according to Ref. [1], the set of all possible couplings for (7) can be used to characterize various constraints imposed on the joint distributions of 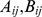 in (8).

From the point of view of all possible couplings, the noncontextuality assumption leading to the single-indexation of the spins, 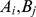, is equivalent to imposing an *identity coupling* on the double-indexed outputs in (7), i.e., creating a coupling (10) – (11) with the additional constraint
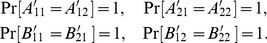
(12)


The Bell-type theorems [13]–[16] tell us that this coupling exists if and only if both the no-signaling condition is satisfied and the four observable joint distributions of 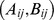 satisfy the inequalities
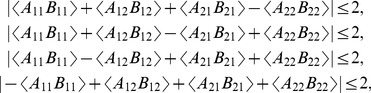
(13)where 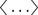 denotes expected value. Clearly, these inequalities do not have to be satisfied, and, in the Alice-Bob paradigm, for some quadruples of settings 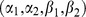, these inequalities are contravened by quantum theory and experimental data.

Therefore, we have to use double-indexing and consider couplings other than the identity coupling (12). This is the essence of the contextualization approach, when applied to the Alice-Bob paradigm. In the conditionalization approach, discussed next, one also uses what can be thought of as a version of double-indexation (*conditioning* on the two indices viewed as values of a random variable), but instead of the couplings in the sense of (10) – (11) one uses a different theoretical construct, *conditional couplings*.

### Conditionalization and Conditional Couplings

One of the simplest ways of creating stochastically unrelated random variables is to consider a tree of possibilities, like this one:
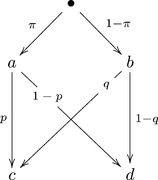
(14)


We have at the first stage outcomes 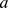 and 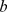, and according as which of them is realized, the choice between 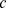 and 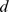 occurs with generally different probabilities. We can consider 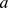 and 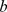 as two mutually exclusive conditions, and use them to label the two random variables
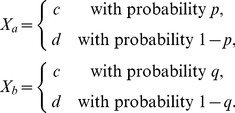
(15)


Clearly, 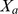 and 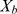 here do not have a joint distribution: e.g., no joint probability 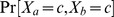 is defined because there is no commonly acceptable meaning in which 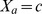 may “co-occur” with 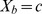. The two random variables here are stochastically unrelated, in conformance with the Contextuality-by-Default principle.

The All-Possible-Couplings principle leads us to consider all joint distributions
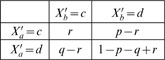
(16)with
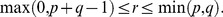
(17)


Each 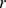 within this range defines a possible coupling 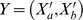 for 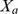 and 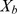. In particular, the independent coupling, with 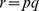, is within the range, while the identity coupling, with 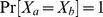, is possible if and only if 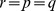.

There is, however, a more traditional view of 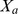 and 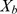 in (14). It consists in considering a joint distribution of two random variables, 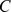 and 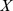, with the marginal distributions
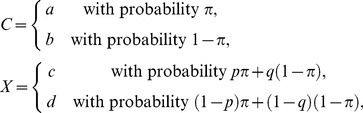
(18)and with the joint distribution
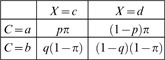
(19)



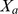
 is then interpreted as 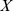 given 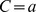, and analogously for 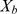. The conditional probabilities are computed as required,
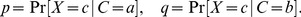
(20)


The idea suggested by this simple exercise is this:

consider any set of stochastically unrelated random outputs labeled by mutually exclusive conditions as if these conditions were values of some random variable, and the outputs were values of another random variable conditioned upon the values of the former.

We call this approach conditionalization. It may seem to provide a simple alternative, within the framework of KPT, to considering all couplings imposable on stochastically unrelated variables. We will argue, however, that this alternative is not theoretically interesting.

Consider the conditionalization of our Alice-Bob paradigm. Denote, for 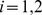 and 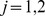,
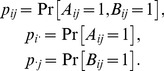
(21)


Introduce a random variable 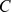 with four values
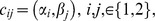
and a random variable 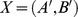 with four values
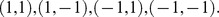
Form the tree of outcomes as shown below, using arbitrarily chosen positive probabilities 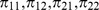 (summing to 1):
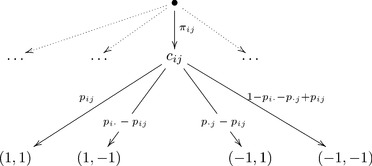
(22)


The conditionalization is completed by computing the joint distribution of 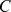 and 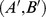:
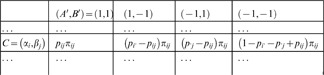
(23)


Clearly, we have constructed a random variable
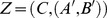
(24)such that
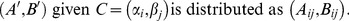
(25)


This 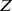 can be called a conditional coupling for 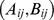, 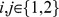.

The conditionalization procedure does not have to claim the existence of any “true” or unique distribution of 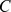. One can freely concoct this distribution, even if the conditions under which 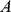 and 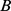 are measured are chosen at will or according to a deterministic algorithm.

There are two interesting modifications of conditionalization, both proposed in a recent paper by Avis, Fischer, Hilbert, and Khrennikov [7]. Instead of the conditional coupling 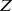 in (24), they consider
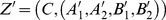
(26)such that
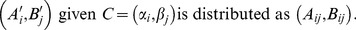
(27)


In other words,
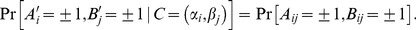
(28)


This does not yet define the conditional probabilities for all possible values of 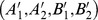. Avis et al. describe two ways of defining them.

In one of them 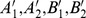 have two possible values each, 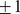, and
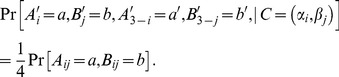
(29)


That is, the probability of 
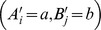
 at 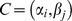 is evenly partitioned among the four values of the “irrelevant” pair 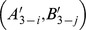. It is easy to see that one could as well use any other partitioning:
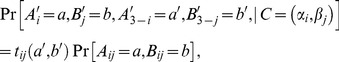
(30)with nonnegative 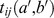 subject to
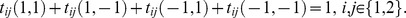



Now, for any distribution of 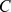 with non-zero values of 
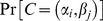
, the joint distribution of 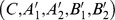 is well-defined.

Another way of implementing (28) described in Ref. [7] is to allow each of 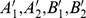 to attain a third value, say, 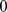, in addition to 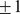. This third value can be interpreted as “is not defined.” It is postulated then that
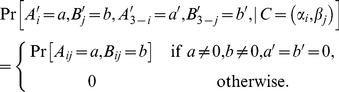
(31)


Again, it is easy to see that the joint distribution of 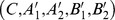 is well-defined and satisfies (28) for any distribution of 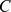 with non-zero values of 
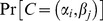
.

## Discussion

### Comparing the Two Approaches

Conditionalization and contextualization achieve the same goal — “sewing together” stochastically unrelated random variables within the confines of KPT. But the similarity ends there. Consider, e.g., the Alice-Bob experiment in which both Alice and Bob use some random generators to choose between two possible measurement directions. Clearly then 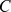 is objectively a random variable, and a joint distribution of 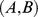 and 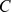 objectively exists. Put differently, in this case 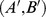 given 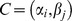 in (25) is simply equal to 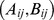.

However, whether 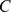 is objectively a random variable or a distribution for the settings is invented, the quantum-mechanical analysis of the situation begins with computing the (conditional) distributions of 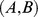 at different settings. The distribution of 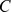 in no way advances our understanding of how 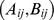 for different 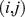 are related to each other.

Thus, we know that the entangled spin-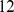 particles are subject to Tsirelson's inequalities [24]

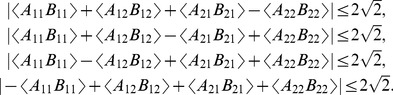
(32)


We also know that if the two particles were not entangled, they would be subject to the Bell-CH-Fine inequalities (13). The difference between these two constraints is not reflected in the “true” distribution of 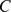, if it exists, nor is it implied by or can in any way restrict the possible choices of “imaginary” distributions of 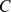. In fact, the only restriction imposed on the distribution of 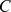, a universal one, is that none of the conditions should have probability zero, because this would make the conditional probabilities undefined. Moreover, the set of possible conditional couplings is the same whether the no-signaling condition is or is not satisfied.

Although in this discussion we assumed that conditionalization was implemented in its simplest version, (24) – (25), our arguments and conclusions apply verbatim to the modifications proposed in Ref. [7] and described at the end of the previous section. The conditional distributions of 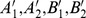 for the four values of 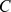 in (29) and (31) are uniquely determined by the observed distributions of the four pairs 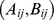. But whatever these distributions, they can be paired with any distribution of 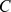, provided none of its values has zero probability.

All of this stands in a clear contrast to the analysis of all possible couplings (10) in the contextualization approach [1]–[4]. In this approach we can ask various questions about the compatibility of couplings with various constraints known to hold for the observable joint distributions. Thus, we may ask about the *fitting set* of couplings for a given constraint (say, Bell or Tsirelson inequalities), i.e., the couplings that are compatible with the spin distributions subject to the constraint. We can also ask about the *forcing set* of couplings, those compatible only with the spin distributions subject to a given constraint. Or we can conjoin the two questions and ask about the *equivalent set* of couplings, those compatible with and only with the spin distributions subject to the constraint. The answers to such questions will be different for different constraints being considered.

Since the four observed joint distributions of 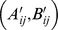 in (11) are themselves part of the couplings (10), the questions above are only interesting if they are formulated in terms of the unobservable parts of the couplings. In the examples below we characterize the couplings in terms of the *connections* [1], [2], [4], which are the (unobservable) pairs
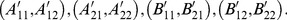
(33)


The diagram below shows the connections in their relation to the pairs whose joint distributions are known from observations (compare with diagram (8)):
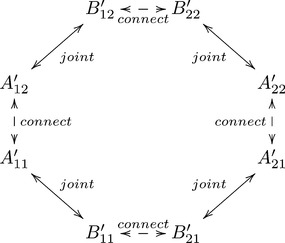
(34)


Let us assume that the probability of spin-up (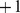) outcome for every (spin-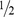) particle in the Alice-Bob paradigm is 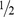. (As shown in Ref. [23], this can always be achieved by a simple procedural modification of the canonical Alice-Bob experiment.) This assumption is, of course, in compliance with the no-signaling condition, which therefore can be omitted from all formulations below.

We know [1] that the following two statements about connections are equivalent:

(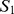) a vector of connections (33) is compatible *with and only with* those distributions of 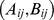, 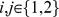, that satisfy the Bell-CH-Fine inequalities (13);
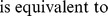

  (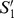) a vector of connections (33) is such that
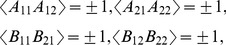
(35)where the number of + signs among the four expected values is 4,2, or 0.

The equivalence of these two statements is an expanded version of Fine's theorem [16], whose formulation in the language of connections is: the identity connections, those with
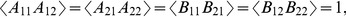
(36)are only compatible with distributions of 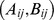 satisfying the Bell-CH-Fine inequalities; and if these inequalities hold, then 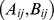 can be coupled by means of the identity connections.

We also know [1] that the following two statements about connections are equivalent:

(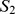) a vector of connections (33) is compatible *with and only with* those distributions of 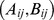, 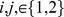, that satisfy the Tsirelson inequalities (32);
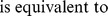
(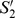) a vector of connections (33) is such that
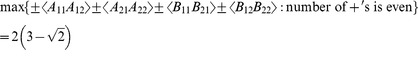
(37)and
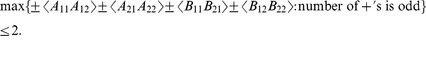
(38)

We see that although the expectations 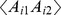 and 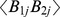 for the connections are not observable, they provide a theoretically meaningful way of characterizing the way in which the stochastically unrelated and observable 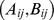 are being “sewn together.” And these ways are different for the Bell-CH-Fine and Tsirelson inequalities.

What can contextualization tell us about the basic predictions of the quantum theory for the Alice-Bob experiment? The theory tells us that, for 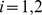 and 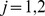,
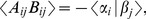
(39)where 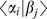 is the dot product of two unit vectors. It can be shown [25]–[27] that the four expectations 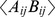 can be presented in the form (39) using a quadruple of setting 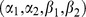 if and only if
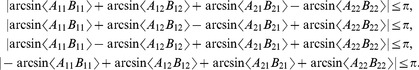
(40)


These inequalities are “sandwiched” between the Bell-CH-Fine ones and Tsirelson ones. That is, they are implied by the former and imply the latter. It is shown in Ref. [4] that

(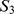) there is *no* vector of connections (33) that is compatible *with and only with* those distributions of 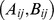, 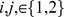, that satisfy the quantum inequalities (40).

Moreover, this negative statement still holds if one replaces the connections (33) with any other subsets of (10), e.g.,
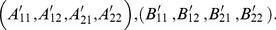
(41)


No distributions of such subsets are compatible with and only with those distributions of 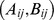 that satisfy the quantum inequalities (40).

The investigation of the forcing set of couplings provides additional insights into the special nature of quantum mechanics. The result we have [4] says that the following two statements about connections are equivalent (note the change from “with and only with” of the previous statements to “only with”):

(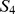) a vector of connections (33) is compatible *only with* those distributions of 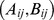, 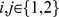, that satisfy the quantum inequalities (40);
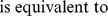
(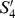) a vector of connections (33) is compatible *only with* those distributions of 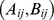, 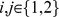, that satisfy the Bell-CH-Fine inequalities (13).

In other words, a choice of connections can force all 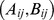 compatible with them to comply with quantum mechanics only in the form of their compliance with classical mechanics.

## Conclusion

The examples just given should suffice to illustrate the point made: while both contextualization and conditionalization embed any input-output relation into the framework of KPT, only contextualization provides a useful tool for understanding the nature of various constraints imposed on the observable joint distributions (one could say also, for different types and levels of contextuality). Conditionalization is uninformative, as any distribution of the conditions is compatible with any distributions of the conditional random variables.
